# Impact of Abandoned Mining Facility Wastes on the Aquatic Ecosystem of the Mogpog River, Marinduque, Philippines

**DOI:** 10.5696/2156-9614-10.26.200611

**Published:** 2020-05-28

**Authors:** Catherine B. Gigantone, Marisa J. Sobremisana, Lorele C. Trinidad, Veronica P. Migo

**Affiliations:** 1 School of Environmental Sciences and Management, University of the Philippines Los Baños, College, Laguna, Philippines; 2 National Institute of Molecular Biology and Biotechnology, University of the Philippines Los Baños, College, Laguna, Philippines; 3 Department of Chemical Engineering, College of Engineering and Agro Industrial Technology, University of the Philippines Los Baños, College, Laguna, Philippines

**Keywords:** mine waste pollution, heavy metals contamination, Marinduque, acid mine drainage

## Abstract

**Background.:**

Mine waste from abandoned mining sites can cause environmental degradation and ecological imbalance to receiving water bodies. Heavy metal pollution affects local communities and may pose health risks to the general public. An abandoned mining facility in Marinduque, Philippines, situated on the of Mogpog River, continuously deposits mine wastes, which may affect the river and the health of local communities.

**Objectives.:**

The aim of the present study was to examine the presence and extent of heavy metal contamination from mine wastes in the aquatic ecosystem of the Mogpog River by determining the level of heavy metal concentration in the water, sediments and biota.

**Methods.:**

Four sampling sites were monitored for heavy metals (copper (Cu), arsenic (As), chromium (Cr) and sulfur (S)) pollution. Several analyses were conducted to determine the heavy metals present in the water, sediment and biota. Atomic absorption spectrophotometry was used for the analysis of Cu concentrations in water. X-ray fluorescence was used for the analysis of total heavy metals in the sediments and biota.

**Results.:**

An inverse relationship with water and sediment from upstream to downstream of the river were observed. This trend shows deposition of Cu in the sediments as factored by pH. Flora gathered from the riverbanks recorded concentrations of Cu in their leaves and fruits.

**Conclusions.:**

It has been difficult for the Mogpog River to regain water quality after years of mine waste deposition. Acid mine drainage occurred upstream of the river which affects the speciation of heavy metals. The potential risk of heavy metal exposure to local communities was observed due to the communities' river utilization.

**Participant Consent.:**

Obtained

**Ethics Approval.:**

The Office of Vice Chancellor for Research and Extension of University of the Philippines Los Baños approved the study

**Competing Interests.:**

The authors declare no competing financial interests.

## Introduction

A previous study has been conducted to determine toxicities of heavy metals to humans and their harmful effects on the environment.^1^ The most significant industries which introduce heavy metals into the environment are mining, coal fired power plants and waste disposal.^1^ Extraction of ore deposits during mining creates large volumes of rock wastes or mine tailings which contain high levels of heavy metals. These waste materials, when released into the environment, are highly toxic and cause adverse effects to the ecosystem and human health.^2,3^ Heavy metals cause histological and morphological changes in tissues, physiology, behavior, reproduction and biochemistry of blood and enzymes.^4^ Human exposure of heavy metals may exhibit acute or chronic illnesses such as *Itai-itai* (cadmium poisoning), Minamata disease (mercury poisoning) and keratosis (arsenic poisoning).^5^

The Philippines is very rich in natural and mineral resources; exploration of mineral resources began as early as the 1900's during American colonization.^6^ Gold and copper are the most abundant mineral resources in the country.^6^ Marinduque, an island in the center of the country, has vast resources of gold containing copper ores. Two mining companies previously operated on the island: Consolidated Mining Inc. and Marcopper Mining Corporation. Both have had incidents of mine tailings disposal in Marinduque's aquatic ecosystems.

In 1993, the Maguila-guila earthen dam collapsed due to heavy rains, depositing large volumes of mine wastes into the Mogpog River, causing adverse environmental impacts to terrestrial and aquatic ecosystems. Health impacts of heavy metal intoxication to nearby communities were reported and the river was declared to be biologically dead.^7^ The present study evaluates the presence and extent of heavy metals, specifically copper (Cu), chromium (Cr), arsenic (As) and sulfur (S) contamination from mine wastes in the aquatic ecosystem of the Mogpog River by determining the levels of heavy metal concentrations in the water, sediments and biota of the river system. Marcopper porphyry copper, a high-grade copper, was mined in Marinduque. Copper speciation in water and sediments spatially distributed in Mogpog River is highlighted. The area is innately rich in Cu, hence it is expected to have high Cu concentrations relative to other trace metals.

**Abbreviations**
LOD: Limit of detection

## Methods

**Figure 1:**
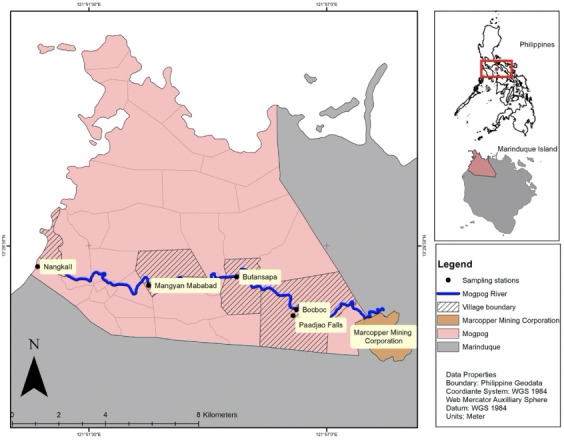
Study sampling sites

Marinduque is an island province close to the center of the Philippines *(Figure 1).* Mogpog is one of six municipalities within Marinduque.

The Mogpog River is a shallow river with a depth ranging from 2–3 feet. It was declared biologically dead due to mine tailings spillage in 1993.^8^ Prior to the disaster, the river was used for laundering clothes, planting water spinach (locally known as *kangkong*), fishing and other recreational activities by local communities. Marcopper Mining Company is situated in a watershed drainage of the Mogpog River. Four sampling points within the Mogpog River were selected based on their proximity to the mining area *(Figure 1).* Bocboc, the first village posterior to the mining site was selected as an upstream sampling site, followed by Butansapa (midstream), Mangyan Mababad (midstream) and Nangka II (downstream). A control site (Paadjao Falls) was also selected based on its proximity to the sampling sites, without being affected by mining activities. Sediments, water and biota samples were collected and analyzed for total heavy metals (Cu, As and S) content.

### Water quality analysis

Temperature, pH, and copper content were analyzed in the four sampling sites. Atomic absorption spectrophotometry was used in the determination of Cu concentration in the riverine water.

### Sediment and biota heavy metal analysis

An X-ray fluorescence spectrophotometer (Niton) was used in the analysis of the sediment and biota's total heavy metal concentration. The Canadian Sediment Quality Guidelines and Standards were used in the analysis of threshold effect levels and probable effect levels in the organisms present in the river.^9^ The Mogpog River remains heavily affected by mine waste leaking from abandoned facilities causing most parts of the river to be biologically dead. It has been difficult for the Mogpog River to regain water quality, which affects the diversity of its flora and fauna. Collated samples were limited to the available flora and fauna species at the time of sampling which resulted in a small sampling size of each species. Only 1 g dried sample per species were yielded from dried composite samples. For example, ~10 guppies yielded ~1 g of dried samples; 3 individual ferns yielded ~1 g dried sample.

### Social survey

A survey on local residents' knowledge, perceptions and attitude was conducted to determine possible exposures of nearby communities along the riverbanks to heavy metals in the river as factored by their current river usage. The total population of the four villages surveyed is 2794 based on 2015 population census data.^10^ Total number of households was estimated at 559. Using Slovin's formula,^11^ one respondent from each of 61 households were considered at 90% confidence level and 10% margin of error. Purposive sampling was used in selecting the respondents of the study. Women aged between 18 to 60 were considered as respondents for the study. Women were chosen as respondents because in general they are more knowledgeable about domestic use of river water for activities such as bathing and laundering. Informed consent was obtained from each participant. Prior to the conduct of the study, approval from the Office of Vice Chancellor for Research and Extension of University of the Philippines Los Baños was obtained.

## Results

**Figure 2:**
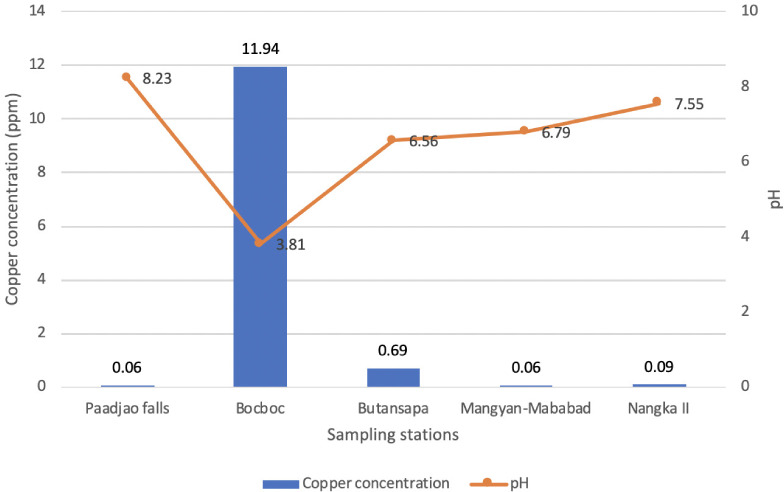
Copper concentrations and pH of riverine water in various sampling sites of the Mogpog River

Figure 2 shows the Cu concentration and pH levels in water from 4 sampling sites along the Mogpog River and a control site, Paadjao Falls. High levels of Cu were present in the riverine water situated at Bocboc (11.936 ppm) compared to the control site (0.06 ppm) and other sampling sites along the whole stretch of Mogpog River (Butansapa 0.69 ppm; Mangyan Mababad 0.06 ppm; and Nangka II 0.09 ppm).

**Figure 3:**
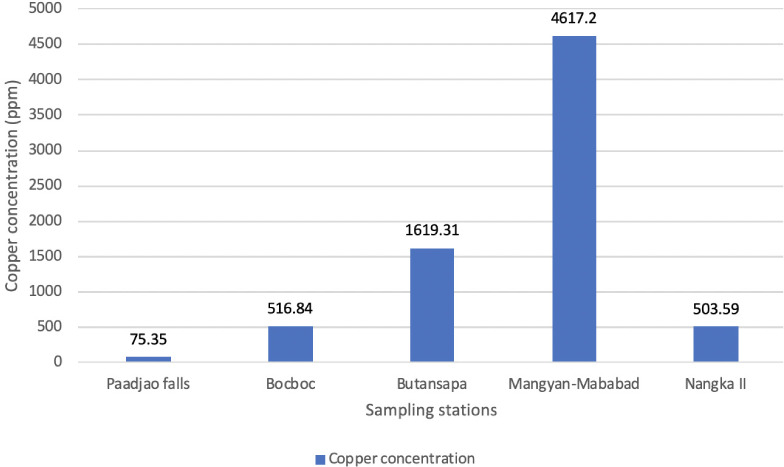
Copper concentration of sediments collected from various sampling sites along the Mogpog River

Figure 3 shows the Cu concentration levels in the sediments from 4 sampling sites along the Mogpog River and the amount of Cu in the control site Paadjao Falls. Mangyan Mababad had the highest concentration of Cu compared to the other three sites.

**Figure 4:**
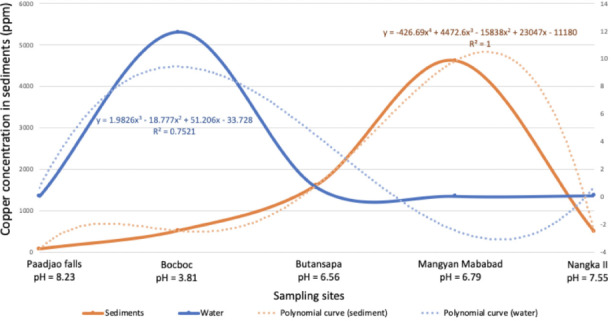
Polynomial regression of copper in sediments and water of the Mogpog River

Further comparison of Cu adsorbed in sediments and dissociated in water using polynomial regression (4^th^ order and 3^rd^ order consecutively) showed an inverse relationship. Most of the Cu coming from the effluents accumulates in the sediments as shown in Figure 4.

### Heavy metals in biota

Table 1 shows the Cu levels in various fauna available along the Mogpog River in comparison to fauna collected at the control site (Paadjao Falls). Fish collected from Bocboc migrated from Paadjao Falls. The sampling area is near the merging of water from upstream of Bocboc and Paadjao Falls. Guppy (Poecilia sphenops) collected from Bocboc had higher levels of Cu (121.19 ppm) compared to guppy samples collected at Paadjao Falls (116.37 ppm). High levels of Cu dissociate in water and changes in pH are some of the externalities (from 8.23 to 3.81), which cause the non-survival of living biota in the Mogpog River situated at Bocboc. Copper concentrations of Poecilia sphenops (guppy) collected from Bocboc station showed 4% more Cu compared to those collected in Paadjao Falls (control station). Only a few fauna species were collected in the sampling sites due to the inability of the organisms to proliferate in the riverine water.

**Table 1:** Copper Levels in Available Fauna in Paadjao Falls, Bocboc and Nangka II

**Sampling station**	**Fauna species**	**Total Cu concentration (ppm)**	**Cu toxicity threshold (ppm)**
Paadjao Falls	Potamalpheops sp. (shrimp)	109.57	15^12^
	Poecilia sphenops (guppy)	116.37	112–138^13^
Bocboc	Poecilia sphenops (guppy)	121.19	112–138^13^
	Megascolex coeruleus (worm)	219.42	n.d.
Nangka II	Pagurus sp. (hermit crab)	1862.89	n.d.

Abbreviation: n.d., no data.

Table 2 shows the flora samples gathered from various sampling stations along the riverbanks of the Mogpog River. Available flora with direct contact to sediment and riverine water were collected and analyzed using X-ray fluorescence analysis. Based on the available data presented in Table 2, very high Cu content was recorded in the flora species situated along the Mogpog riverbanks. This is observed in Nephrolepis biserrata, a fern species, which is found in Paadjao Falls and Bocboc with values of 34.06 ppm and 225.76 ppm, respectively. Another species found to have high levels of Cu was Albizia saman (rain tree) found in Butansapa (49.12 ppm) and Mangyan Mababad (49.59 ppm). Cassia alata gathered from the Nangka II estuary had the highest Cu content (334.58 ppm) recorded among all the sample species. Based on a study conducted by Dutta and Ghosh, Cassia alata is a hyperaccumulator plant species.^14^ Generally, the results of the present study suggest that the streams of the Mogpog has high bioavailable Cu in its sediments and water; and species found along the riverbanks are tolerant of high concentrations of Cu.

**Table 2:** Copper Concentrations in Various Flora Species Gathered from Designated Sampling Sites along the Mogpog River

**Sampling stations**	**Flora species**	**Total Cu concentration (ppm)**
Paadjao Falls	Nephrolepis biserrata	34.06
	Justicia gendarussa	46.73
Bocboc	Nephrolepis biserrata	225.76
	No ID	52.83
Butansapa	Amphineuron sp.	118.79
	Albizia saman	49.12
Mangyan Mababad	Albizia saman	49.59
	Pityrogramma calomelanos	133.9
Nangka II	Cassia alata	334.58
	Quisqualis indica	85.86

Collected flora species were selected based on availability at the sampling stations. No indicator species were present across all sampling stations. This is due to the altered landscape and toxicity of sediments and water to the biota. Percent differences in Cu concentrations of some species were determined. Nephrolepis biserrata collected from Bocboc station had 85% more Cu compared to those collected in Paadjao Falls (controlled station). Albizia saman collected from Mangyan Mababad had 1% more Cu compared to samples from Butansapa. No literature is available for cross-referencing the level of Cu in these flora species.

Among all the collected flora, only Pityrogramma calomelanos, commonly known as silver back fern, recorded As concentrations, amounting to 36.32 ppm. All sediments tested for As yielded lower than the limit of detection (LOD) of the X-ray fluorescence, a limitation of the instrument used for analysis.

### Other relevant heavy metal in sediments

**Figure 5:**
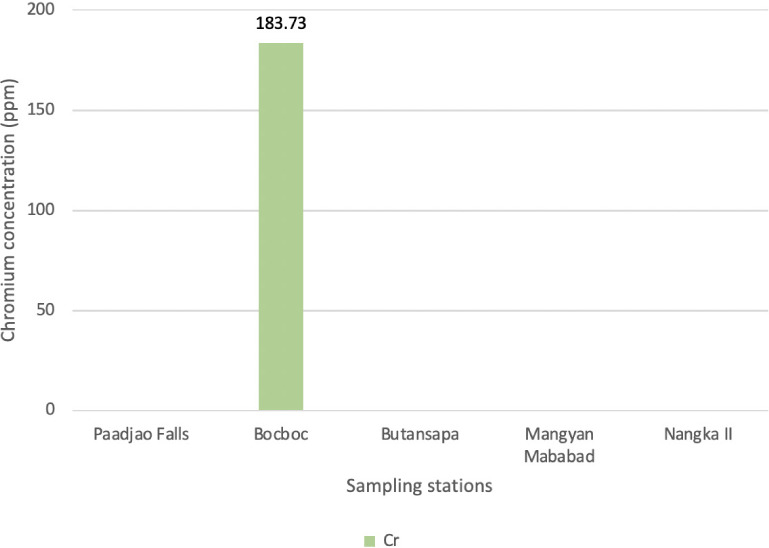
Chromium concentrations of sediments collected from various sampling sites along the Mogpog River

Very high levels of Cr were recorded in Bocboc, compared to Paadjao Falls and other sampling sites which recorded <LOD, as presented in Figure 5.

**Figure 6:**
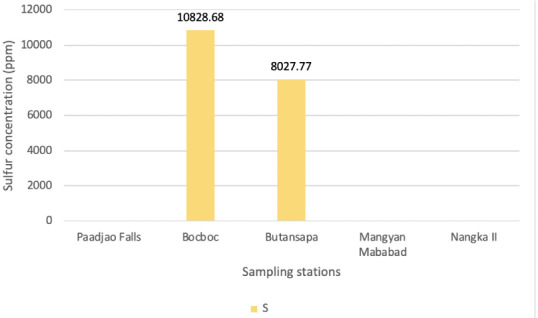
Sulfur concentrations of sediments collected from various sampling sites along the Mogpog River

Figure 6 shows S levels in sediments along the Mogpog River. Very high S concentrations were recorded in Bocboc and Butansapa at 10828.68 ppm and 8027.77 ppm, respectively. High concentrations of S in sediments indicate occurrence of acid mine drainage upstream of the Mogpog River.

### Community exposure to heavy metals

**Figure 7:**
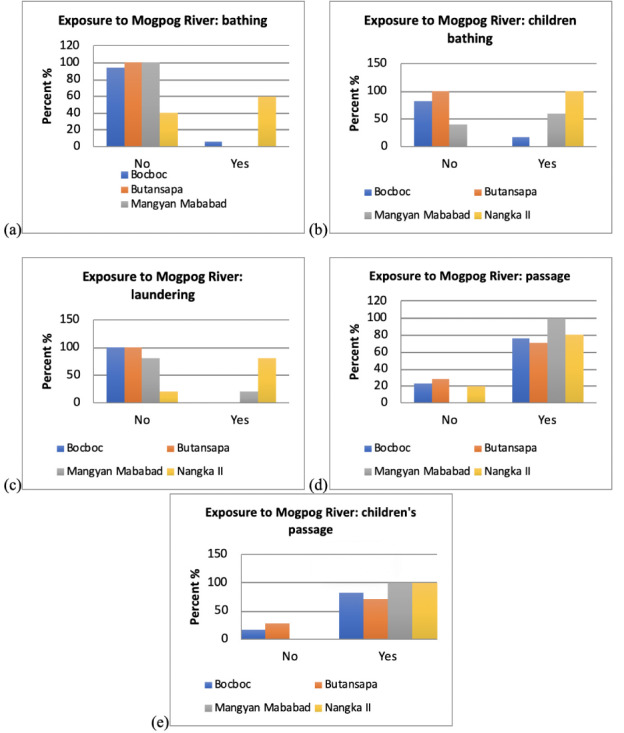
Exposure to Mogpog River through the following activities: (a) bathing; (b) children bathing; (c) river crossing; (d) children's river crossing; and (e) laundry

Figures 7 a–e present the respondents' self reported exposures in several communities along the Mogpog River. Respondents from upstream and midstream villages of Mogpog do not use the river for laundering and bathing, but frequently cross the Mogpog River, while respondents from Nangka II still use the Mogpog River for laundering and bathing. The children in villages upstream of the Mogpog River do not bathe in the river. All of the children across all sites were observed to cross the river more frequently compared to adults.

## Discussion

In general, cupric ion (Cu^2+^) was the dominant copper species in conditions with pH 3.81, while precipitates of copper hydroxide (Cu(OH)_2(s)_) and Cu^2+^ were present at pH 6.5 – 7.55.^15^ It can be inferred that in the river, the dominant Cu species present was in the form of dissociated Cu^2+^, as observed by the high Cu concentration in Bocboc (11.9360 ppm at pH 3.81) riverine water. Similarly, the decline in Cu concentration observed in Butansapa (0.68682 ppm at pH 6.56) and Mangyan Mababad (0.05605 ppm at pH 6.79) shows changes in the state of Cu. The neutral (6.5–7.55) pH of water caused the formation of Cu(OH)_2(s)_. Comparing the values for dissolved Cu and pH to standards set by Department of Environment and Natural Resources, Administrative Order 34 and 2016–08, the riverine water of the Mogpog River from Bocboc to Nangka II exceeded the acceptable level of dissolved Cu (Class AA–C 0.02 ppm; Class D 0.04 ppm) and Bocboc (pH 3.81) exceeded the acceptable pH level for Class AA–B (6.5–8.5) and Class C–D (6.0–9.0).^16,17^

**Figure 8:**
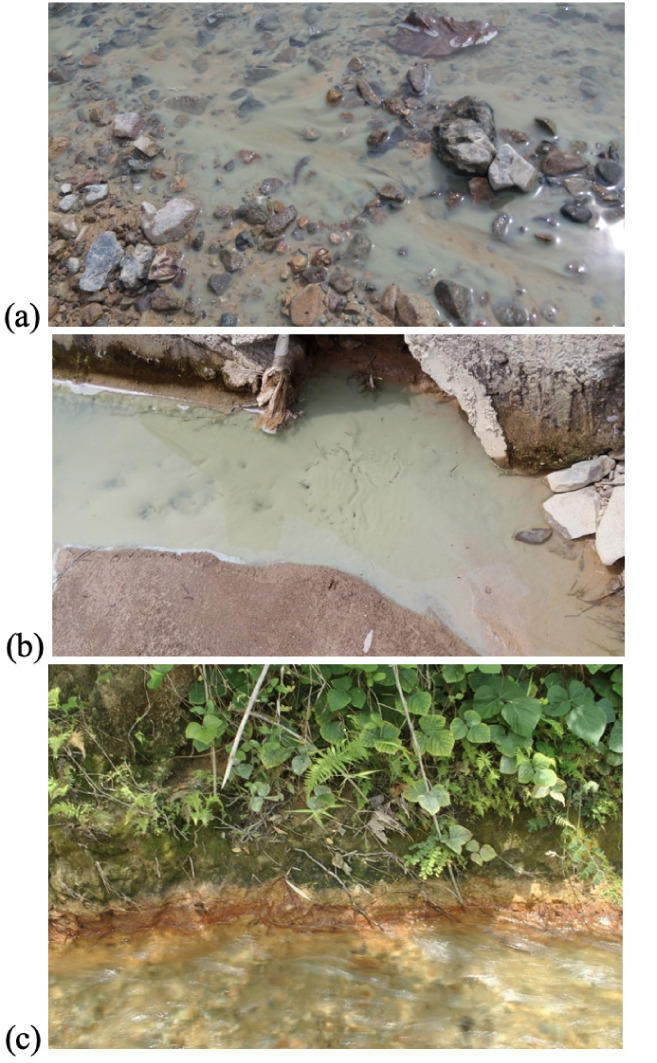
Documentation of mine waste pollution in Bocboc, Mogpog River sediments (a & b) suspended milky-white colloids; and (c) rust deposits along the riverbank

The control site Paadjao Falls had Cu levels exceeding the Administrative Order 2016–08 standard for water quality.^17^ This data suggests that Marinduque is naturally rich in Cu. Comparison of Cu content in Bocboc (11.94 ppm), Butansapa (0.69 ppm) and Nangka II (0.09 ppm) to Paadjao Falls (0.06 ppm) shows that Cu content in the river stream is relatively high. It can be inferred that there is continuous leakage of wastewater and sediments coming from the Marcopper Mining Corporation into the Mogpog River, confirming the observation of local residents, as shown in Figure 8. The Bocboc site had an acidic pH that may be an indication of acid mine drainage in the area due to mine wastewater and sediments from abandoned mining facilities. This is brought about by the reaction of mined sulfide rocks with dissolved and/or atmospheric oxygen. The pH levels affect the speciation of heavy metals in water causing the decreasing trend of Cu in water as observed downstream.^18^ On the other hand, increases in the concentration of Cu from 0.05605 ppm (Mangyan Mababad) to 0.09145 ppm (Nangka II) may be brought about by salinity of the estuary wherein dissolution of Cu precipitates (Cu_2_O_(s)_) to aqueous forms of copper chloride (CuCl_(x)(aq)_) is facilitated.^19^

### Copper in sediments

There was an increasing trend in heavy metals concentration in sediments moving further downstream. This trend can be attributed to the pH of the riverine water, which influences the fate of Cu: either it can be dissociated in water in ionic form (acidic); or it forms precipitates and adheres to sediments (neutral to basic).^15^ In this case, Cu precipitated from water and adhered to sediments due to its pH value (6.56 to 6.79). A decline in Cu concentration in sediments gathered in Nangka II, an estuary, may be attributed to mixing of water during wave movement and tide influx. As pH increases under saline conditions, Cu precipitates (Cu_2_O_(s)_) dissolve forming Cu-chloride (Cl) complexes.^19^ This is evident in the sudden drop of Cu concentration from 4617.2 ppm (Mangyan Mababad) to 503.59 ppm (Nangka II), although relatively high compared to 75.35 ppm recorded in Paadjao Falls. Comparing the natural Cu present in soil, represented by sediments in Paadjao Falls, there is a significant increase in the Cu concentration of sediment along the Mogpog River.

### Copper in sediment-water interaction

As observed in Figure 2 (Cu in water) and Figure 3 (Cu in sediments), there was an increasing level of heavy metals present in the sediments downstream of the river, but decreasing levels of heavy metals dissociated in the water.

This relationship is illustrated in Figure 4. It can be inferred that the dominant species of Cu was Cu^2+^ in Bocboc brought about by low pH value (3.81). On the other hand, dominant Cu speciation at Butansapa and Mangyan Mababad was solid Cu(OH)_2_ due to its neutral pH (6.56 and 6.79, respectively).^15^

The earthen dam (Maguila-guila dam) drains to the Mogpog River. Bocboc is the nearest sampling station in the upstream part of the river. Sedimentation was observed in the area which caused shallowing of the river (2–3 feet depth).

### Other heavy metal in biota

Silver back fern (Pityrogramma calomelanos) is a metallotolerant species specific to As and is an As hyperaccumulator.^20^ Ferns are observed to thrive in Cu enriched substrates.^20^ Other fern species (Nephrolepis biserrata and Amphineuron sp.) gathered from Paadjao Falls, Butansapa and Bocboc recorded values of As less than LOD (9 ppm). This can be attributed to the specificity of plants to accumulate or hyperaccumulate certain heavy metals. Extremophiles are important in ecological assessments as they can be used for monitoring the extent of pollution in the environment.^21^

### Heavy metals in sediment

High concentrations of detected Cr indicate that the mine wastewater and sediments contain Cr, thus contaminating Bocboc sediments. Low readings of Cr in other sampling sites may imply that Cr from upstream may have been accumulated in the biota or changed its speciation. The Cr present in the sediments may be present as organic salts, which is bioavailable for organism uptake. Various flora gathered across sampling sites had <LOD (65 ppm) levels of Cr. It can be concluded that these species are not accumulators, but can withstand stressful conditions such as high concentration of heavy metals (Cu, Cr, lead, iron, stannum, etc.).

Among all the heavy metals, only Cr showed a strong negative correlation (−0.97) using the Pearson correlation (with 0.034 significance) with respect to the pH of riverine water. This indicates that Cr deposition is highly affected by pH. Other heavy metals were detected with positive correlation but low statistical significance.

High concentrations of S in Bocboc and Butansapa indicate that the rocks in the area are rich in S. When rocks or sediments containing high S concentration come into contact with water in the form of rain, run-off or river water, acid mine drainage occurs. The Marinduque geological composition consists of chalcopyrite, pyrite and magnetite, among others.^22^ When pyrite, an iron sulfide, comes in contact with water, sulfuric acid and iron deposits are formed.^23^ This caused acidic river water in Bocboc and slightly acidic water in Butansapa. Water pH determines the speciation of heavy metals.

## Conclusions

The Mogpog River has had difficulty in reversing habitat loss since a mining incident in 1993. The continuous discharge of mine wastes impedes the natural succession of the aquatic ecosystem. Lack of aquatic biota suggests that the mine waste discharge is toxic and poses lethal effects to the aquatic ecosystems of the Mogpog River.

Results of the study showed that continuous outflow of mine waste still affects the Mogpog River. The present study recorded significantly high levels of Cr, Cu and S in sediments and biota, among other heavy metals, compared to the control site. Acid mine drainage occurs in Bocboc, as indicated by its low pH. The level of pH affects the speciation and bioavailability of heavy metals in water and sediments and is also a factor in the recuperation and succession of aquatic life in the river.

### Recommendations

Regular monitoring and assessment of water quality, sediment transport and ecological impacts along the Mogpog River must be conducted to determine the ecological recuperation of the aquatic ecosystem and the externalities affecting it. Copper speciation and bioavailability must be conducted to determine possible threats to local communities. In-depth studies on the pathways of heavy metals (air, water and soil) and effects on these communities is also recommended. Infrastructure risk assessment of the abandoned mining facilities must be conducted to carry out rehabilitation of infrastructure (i.e. dams and tailing pits) and prevent potential hazards to the communities. There is a need to address pollution sources for effective rehabilitation of the area.
